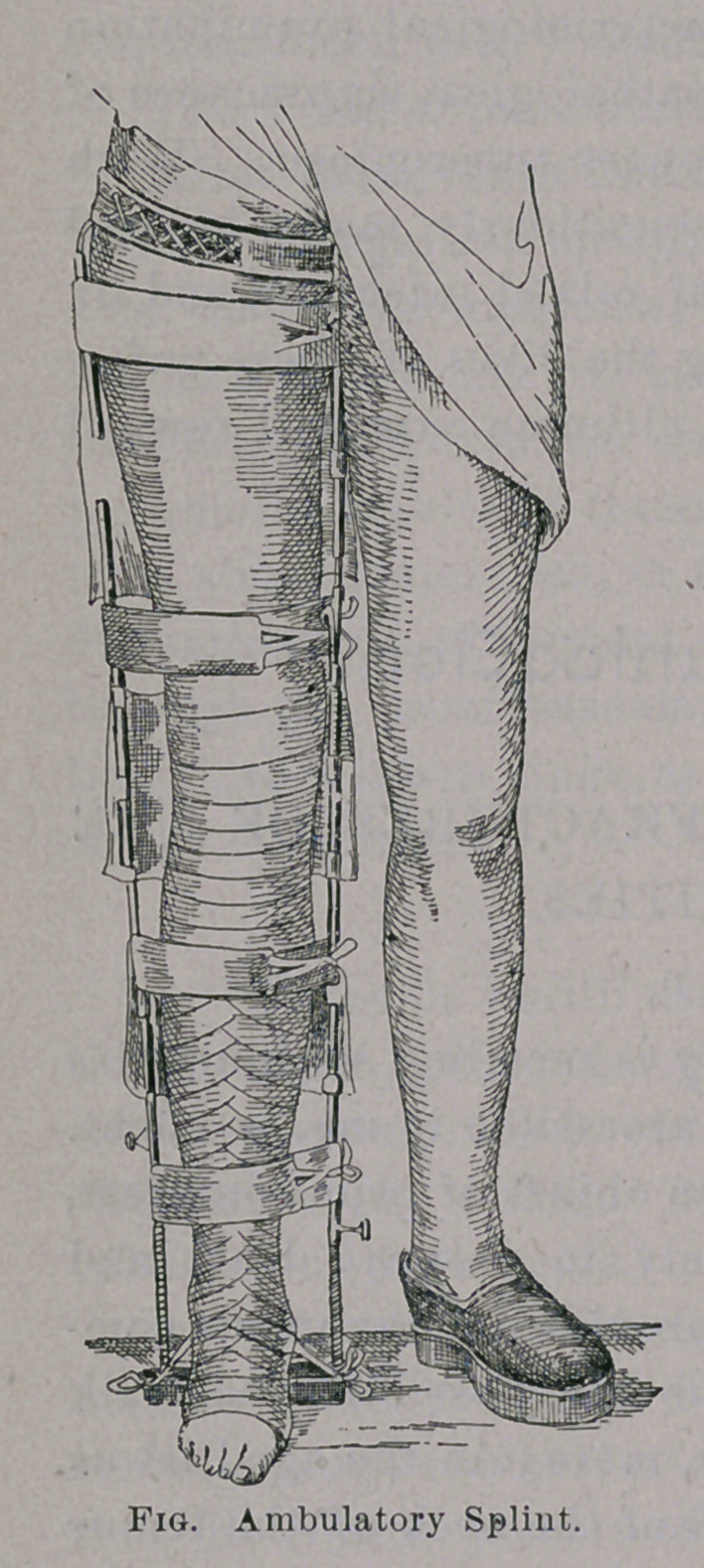# Ambulatory Treatment in Fractures of the Lower Extremities1Read at the Surgical Section, Buffalo Academy of Medicine, October 2, 1894.

**Published:** 1894-11

**Authors:** M. Hartwig


### Original Communication.

### AMBULATORY TREATMENT IN FRACTURES OF THE LOWER EXTREMITIES.[1]

By M. HARTWIG, M. D.

Whilst the German literature of the day is teeming with articles about this subject, the English, readily accessible to me, is silent. It is certainly to the scientific mind not an object of prime interest, but to the patient. Our duty is not only to prevent death and suffering, but wherever we can to even provide for luxurious comforts of our patients. Such certainly is the possibility to walk around with a broken leg or thigh. Nay, more; in the aged let us say, with a fracture high up, for example of the neck of the femur which may require three months' treatment, it is life-saving to have an appliance which permits walking, because it prevents the death-dealing hypostatic pneumonia.

Thus, the object is worthy of consideration without being a novelty, and we can understand the glowing expression of thanks bestowed by the well-known clinician, Professor Juergensen, upon Mr. Hessing, when the former broke his thigh and the latter enabled him to perambulate in the garden with his fracture.

1. Read at the Surgical Section, Buffalo Academy of Medicine, October 2, 1894.

Here is one of the exceptional cases in the history of medicine where a layman distinctly promoted the art of healing, and it is an honor to the profession that the man found his acknowledgments. The name of this instrument-maker will remain honored in the annals of medicine. That there is nothing new under the sun remains true in philosophy, but never in the realm of natural science, and even if a scrupulous search in the annals of medicine should disclose that somebody walked with a broken leg (and I have no time to institute such a search today), Hessing's work will remain a new blessing to suffering mankind—the principle he probably caught in making Taylor's splint for hip-joint disease.

FIG. Ambulatory Splint.

Herewith almost the whole of it is said. The patient sits on the tuber schii which forms the point of counter-extension, while the extension is on the foot toward a crossbar on the sole of the apparatus. The sole of the other leg is raised by a cork nailed to the shoe. Bruns modified the apparatus by making it extensible from 52 to 72 cm. for children, and from 72 to 92 for men. W. Benerle, in Tübingen, furnishes the large size for 23 marks, weighing only a little over two pounds. (See Fig.)

Frequently counter-extension is unnecessary, the weight of the leg being sufficient. Within the apparatus, of which I hand you a self-explanatory picture, the place of the fracture, and, to some extent, above and below is encircled by a very light plaster cast laid upon the bare, oiled and shaved skin.

My quondam friend Albers, staff physician of the Prussian army, has obtained the same result by plaster casts alone, which he makes so deftly and strong as to carry the body without breaking, and without being to weighty, by combining the plaster with glue and tough woodstrips, and including the hip up to the crista ilei. The plaster casts, in order to fit snugly, have to be applied after the original swelling has subsided. Thus, about a week after the fracture occurred, the patient walks on the cast. It projects beyond the sole of the foot and has a space between it and the sole. The same apparatus is used successfully by Bruns for resection of the knee. The gentle physiological stimulus of walking has been far from preventing solid union; the reverse, in fact, seems to be true, as ununited fractures left after such treatment have not been reported yet—not to my knowledge at least.

## Figures and Tables

**Fig. f1:**